# A Simulation Study Comparing Different Statistical Approaches for the Identification of Predictive Biomarkers

**DOI:** 10.1155/2019/7037230

**Published:** 2019-06-13

**Authors:** Bernhard Haller, Kurt Ulm, Alexander Hapfelmeier

**Affiliations:** Technical University of Munich, School of Medicine, Institute of Medical Informatics, Statistics and Epidemiology, Ismaninger Str. 22, 81675 Munich, Germany

## Abstract

Identification of relevant biomarkers that are associated with a treatment effect is one requirement for adequate treatment stratification and consequently to improve health care by administering the best available treatment to an individual patient. Various statistical approaches were proposed that allow assessing the interaction between a continuous covariate and treatment. Nevertheless, categorization of a continuous covariate, e.g., by splitting the data at the observed median value, appears to be very prevalent in practice. In this article, we present a simulation study considering data as observed in a randomized clinical trial with a time-to-event outcome performed to compare properties of such approaches, namely, Cox regression with linear interaction, Multivariable Fractional Polynomials for Interaction (MFPI), Local Partial-Likelihood Bootstrap (LPLB), and the Subpopulation Treatment Effect Pattern Plot (STEPP) method, and of strategies based on categorization of continuous covariates (splitting the covariate at the median, splitting at quartiles, and using an “optimal” split by maximizing a corresponding test statistic). In different scenarios with no interactions, linear interactions or nonlinear interactions, type I error probability and the power for detection of a true covariate-treatment interaction were estimated. The Cox regression approach was more efficient than the other methods for scenarios with monotonous interactions, especially when the number of observed events was small to moderate. When patterns of the biomarker-treatment interaction effect were more complex, MFPI and LPLB performed well compared to the other approaches. Categorization of data generally led to a loss of power, but for very complex patterns, splitting the data into multiple categories might help to explore the nature of the interaction effect. Consequently, we recommend application of statistical methods developed for assessment of interactions between continuous biomarkers and treatment instead of arbitrary or data-driven categorization of continuous covariates.

## 1. Introduction

For medical decision making, predictive biomarkers play an important role for various diseases [1–4]. A biomarker is called “predictive,” if the difference between the effectiveness of two or more treatment options depends on the value of that biomarker [5, 6]. In the presence of a qualitative biomarker-treatment interaction [7], i.e., when the choice of the “optimal” treatment for a given patient depends on the patient's value of a certain biomarker, the biomarker can be used for treatment stratification [8]. Biomarkers used in clinical practice for treatment stratification are, e.g., the human epidermal growth factor receptor 2 (HER-2) status for breast cancer patients [9, 10] or presence of epidermal growth factor receptor (EGFR) mutation in non-small cell lung cancers (NSCLC) [11]. Consequently, the identification of biomarkers that allow prediction of the treatment effect when different treatment options are available is essential to increase clinical decision making in the sense of a stratified or personalized medicine [12].

In practice, investigation of such treatment effect heterogeneity over the range of a certain biomarker in data obtained from a randomized clinical trial is often performed by subgroup analyses [13], where the difference in outcome between the study groups, quantified, e.g., by a hazard ratio, an odds ratio, or a mean difference, is estimated for patient subgroups with similar characteristics [14] and compared using a statistical test for interaction, which can be performed by including the product of the biomarker and the variable indicating treatment allocation in an appropriate regression model [15, 16]. While this procedure is intuitive and straightforward for categorical variables, e.g., gender or presence of comorbidities as diabetes, investigation of treatment effect heterogeneity with respect to continuous variables, e.g., age or continuously measured blood parameters, requires categorization of the variable, when subgroup analyses are to be performed. Such categorization of continuous variables was criticized due to loss of information leading to a loss of power for detection of true interactions, implication of biological implausible effects, and lack of comparability of results from different studies [17, 18]. Therefore, various approaches were proposed in the literature that allow to model and test for treatment effect heterogeneity over the range of a continuous variable that do not require categorization of the variable [19–21].

In this article, we describe a simulation study comparing different approaches for detection of an interaction between one (predefined) continuous covariate and treatment. We simulated data as they would be expected to be collected in a randomized clinical trial intended to compare efficacy of two treatment groups or of treatment versus placebo. Consequently, patients are allocated randomly into one of two treatment arms and the distribution of the variable of interest (often referred to as a biomarker [22]) is expected to be the same for both treatment groups. As most predictive biomarkers were identified for treatment of cancer [23], a time-to-event outcome is considered, as typically overall survival or progression-free survival is considered as primary endpoint in randomized phase III oncological trials [24]. Results obtained by methods relying on categorization of the continuous variable as well as methods that do not use such categorization were investigated. We considered a method splitting the continuous biomarker at its median to determine two subgroups for further analysis, the use of four subgroups determined by splitting data at the quartiles, and use of an “optimal” cutoff value found by maximization of the Wald statistics of the interaction term in a Cox regression model. Additionally, we applied the Subpopulation Treatment Effect Pattern Plot (STEPP) approach that incorporates overlapping subgroups [25], the Cox regression model [26] assuming a linear covariate-treatment interaction term, the Multivariable Fractional Polynomials for Interaction (MFPI) approach that incorporates nonlinear transformations for the interaction term [19], and the Local Partial-Likelihood Bootstrap (LPLB) that uses local estimates of the treatment effect at different values of the variable of interest [27]. Different scenarios with absence and presence of biomarker-treatment interactions were investigated in order to estimate and compare type I error probability and statistical power of the different approaches under the given scenarios. Sample size and censoring distribution are varying to investigate the impact of these characteristics on the outcome.

The article is organized as follows. In Section 2, the simulation study is described. The different methods used for identification of a biomarker-treatment interaction are shortly introduced in Section 2.1, and references to original articles and further articles including more detailed descriptions of the considered methods are given. The setting of the simulation study and the relevant aspects that were varied are described in Section 2.2. Results of the simulation study, namely, observed type I error probabilities for scenarios with no true biomarker-treatment interactions and estimates for statistical power for scenarios with truly present biomarker-treatment interactions, are presented in Section 3. A discussion of the results with concluding remarks and strengths and limitations of our simulation study is given in Section 4.

## 2. Methods

The methods investigated in the simulation study are described in Section 2.1. Details on the settings used in the simulation study and the data generating process are given in Section 2.2. Data were generated and analysed using the statistical software R [28]. Cox regression was performed using the function *coxph* provided in the R library *survival* [29, 30]. For convenience, the continuous covariate of interest will be called “biomarker” and denoted as *Z* throughout the section. Treatment allocation will be represented by a binary treatment variable *T* with *T*={0,1}, where *T*=1 represents, e.g., an experimental treatment and *T*=0 a placebo control or standard treatment. As it appears to be the most relevant effect size in practice, homogeneity of the hazard ratio between the study groups in regard to the biomarker of interest was investigated. For all statistical tests, a significance level of *α*=5% was used. Exact 95% confidence intervals for rejection probabilities were calculated.

### 2.1. Methods Used to Test for a Biomarker-Treatment Interaction

#### 2.1.1. Median Split

In many applications investigating treatment-effect heterogeneity in regard to a continuous biomarker, individuals are divided into two subgroups of equal size. This is achieved by splitting the data at the median of the biomarker *Z*. This procedure will be denoted as “Median split” in this article. A binary indicator variable that is assigned the value of one if the biomarker value is above or equal to the observed median and zero else is derived. To test for biomarker-treatment interaction, a Cox regression model with this indicator variable, the binary treatment indicator, and their product (the interaction term) is fitted to the data. The *p* value of the Wald test for the interaction term was used to decide whether the null hypothesis of no biomarker-treatment interaction can be rejected on the prespecified significance level of *α*=5%.

#### 2.1.2. Quartile Split

As an alternative approach, individuals were divided into four subgroups with splits at the corresponding quartiles of the biomarker of interest (“Quartile split”). The categorical variable indicating the corresponding subgroup was used as a dummy coded nominal independent variable in a Cox regression model. Additionally, the binary treatment indicator and an interaction term between the dummy coded categorical variable indicating the biomarker quartile and treatment were included. A likelihood ratio test with three degrees of freedom provided in the R library *car* [31] was performed to test for presence of a biomarker-treatment interaction.

#### 2.1.3. “Optimal” Split

For this approach, henceforth called “Optimal split”, an “optimal” cutoff value for splitting the continuous variable into two subgroups was determined in a first step. Of all possible cutoff values (restricted to a minimum subgroup size of 10% of the overall sample size), the one leading to the largest value of the Wald statistic for the interaction term between the dichotomized biomarker and treatment in a Cox regression model also including the corresponding main effects as independent variables was used to define the subgroups for assessment of treatment effect heterogeneity. In a second step, these subgroups were treated as if they were predefined subgroups, and assessment of a biomarker-treatment interaction was performed as described for the Median split procedure in Section 2.1.1.

#### 2.1.4. Subpopulation Treatment Effect Pattern Plot (STEPP) Method

The Subpopulation Treatment Effect Pattern Plot (“STEPP”) method was proposed by Bonetti and Gelber [25]. In the STEPP procedure, heterogeneity of the treatment effect over the range of a biomarker of interest is assessed by estimating the effect in multiple overlapping subgroups. Additionally, methods for estimation of simultaneous confidence intervals and for testing the null hypothesis of no biomarker-treatment interaction were developed [25, 32]. Two different versions, a “tail-oriented” and a “sliding window” approach, were proposed initially. In our simulation study, we used the “sliding window” approach, where the number of individuals within two consecutive subgroups is held (approximately) constant by adding and eliminating the same number of observations and the number of observations overlapping between two consecutive subgroups is chosen a priori. For our analysis, the number of individuals within each subgroup was chosen to be *n*/5 and the number of overlapping individuals to be *n*/10. So, the subgroup sizes were 50, 100, and 200 for scenarios with 250, 500, and 1000 observations, and the number of overlapping observations was 25, 50, and 100, respectively. This led to a total number of nine subgroups considered irrespective of the sample size. A test on homogeneity of the hazard ratio over all subgroups was performed to test for a biomarker-treatment interaction. A permutation test as recommended in [32] was conducted using 500 permutations for each simulated dataset. Further details on the STEPP procedure can be found in [33, 34]. For application of STEPP, the R library *stepp* [35] was used.

#### 2.1.5. Cox Regression Model with Linear Interaction

To avoid categorization of the continuous biomarker of interest *Z*, a Cox regression model [26] assuming a linear interaction between *Z* and treatment *T* was considered. This procedure implies that the log-hazard ratio between the study groups is linearly associated with the biomarker value. The main effects of the biomarker *Z*, the treatment group *T*, and their product *Z* × *T* were used as independent variables in a Cox regression model. The *p* value of the Wald test for the interaction term was considered to decide on rejection of the null hypothesis of no biomarker-treatment interaction. This procedure will be called “Cox model with linear interaction” or shortly “Cox (linear Int.)” throughout the article.

#### 2.1.6. Multivariable Fractional Polynomials for Interaction (MFPI)

To allow for nonlinear interaction terms, Royston and Sauerbrei proposed the Multivariable Fractional Polynomials for Interaction (“MFPI”) approach [19], which is based on the Multivariable Fractional Polynomials (MFP) approach presented by Royston and Altman [36]. A nonlinear transformation is considered for the biomarker of interest, and a model including main effects of treatment and the transformed biomarker as well as their interaction is compared to a model including only the corresponding main effects. In the original publication, a model with two polynomial transformations *p*_1_ and *p*_2_ (FP2) out of the set *p* ∈ {−2, −1, −0.5, 0,0.5, 1,2,3}, where *p*=0 indicates a logarithmic transformation, was described. Identification of the best transformation was proposed to be determined in the model without an interaction term by finding the combination of transformations providing the highest (log-)likelihood value (later called flex1 approach). Based on the results of a simulation study [37, 38] considering a continuous outcome, an alternative approach with only one polynomial transformation (FP1) and separate determination of the best transformation in the model with and without interaction (flex3, potentially leading to nonnested models) was recommended. We applied both approaches, the FP2-flex1 and the FP1-flex3 approach, to our simulated data. To test for presence of a biomarker-treatment interaction, likelihood ratio tests comparing the models with and without interaction terms were performed for both strategies.

#### 2.1.7. Local Partial-Likelihood Bootstrap (LPLB)

Another method proposed in the literature for modelling nonlinear interaction effects between a continuous biomarker and treatment is the Local Partial-Likelihood Estimation proposed by Fan et al. [21]. Liu et al. developed a bootstrapping method, called Local Partial-Likelihood Bootstrap (“LPLB”), that allows to test for the presence of an overall treatment effect and to test whether the treatment effect is heterogeneous over the range of a continuous biomarker [27]. In the LPLB approach, linear approximations of the treatment effect estimate at a given biomarker value are obtained by first-order Taylor approximations using weighted data in the local neighbourhood of the biomarker value of interest. The proposed bootstrap test makes use of the residual bootstrap [39]. The obtained local estimates of the log-hazard ratio are compared to the estimate obtained from a standard Cox regression model assuming a constant treatment effect over the biomarker range. The maximum observed standardized difference of the local estimates to the constant log-hazard ratio is considered as test statistic. For our simulation study, we used the R library *lplb* [40] to apply the LPLB procedure. Local estimates were obtained for every decile of the empirical biomarker distribution. A bandwidth, indicating the amount of observations in the neighbourhood used for local estimation, of 0.2 was used and an Epanechnikov kernel was considered for weighting. Five hundred bootstrap samples were drawn for each generated dataset.

### 2.2. Simulation Settings

Data were generated to mimic data observed in a randomized clinical trial primarily intended for comparison of two different treatment options. Consequently, simulated individuals were randomly allocated to one of two treatment groups (*T*={0,1}) with equal probability for each group. The covariate of interest was randomly generated from a uniform distribution with a minimum value of zero and a maximum value of one. Event times were drawn from an exponential distribution with the individual hazard rate depending on the allocated treatment group and the drawn covariate value as described in Section 2.2.1. Censoring times were drawn from exponential distributions with rates as described in Section 2.2.3. The lower value of the two time variables was allocated as observed time and an observed event was indicated, if the drawn event time was smaller than the corresponding censoring time, and a censored observation was indicated else.

#### 2.2.1. Functional Form

**Figure 1:**
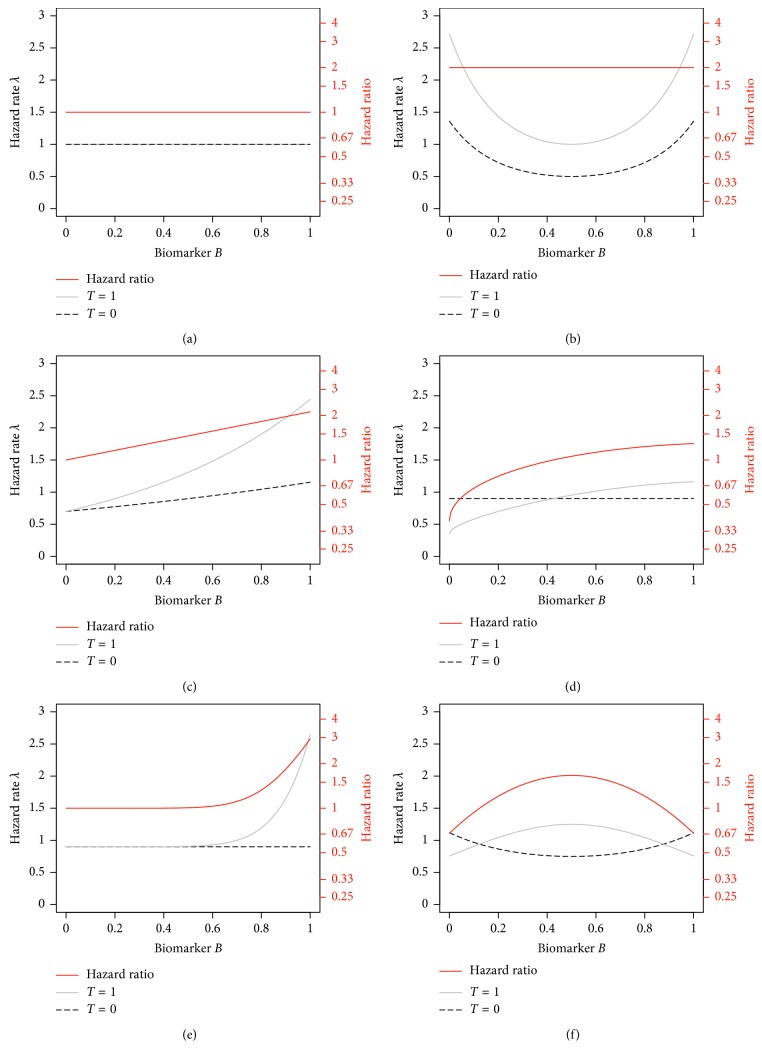
Scenarios used in the simulation study for comparison of statistical methods. In scenarios 1 and 2 (a, b), data are generated under the null hypothesis of no biomarker-treatment interaction. In scenarios 3 to 6, which are illustrated in (c) to (f), the hazard ratio (illustrated on a log-scale by the red line) depends on the biomarker value, so biomarker-treatment interactions are present.

In order to estimate the type I error probability and the statistical power for detection of truly present interaction effects associated with the different approaches, different scenarios were investigated. Overall, six different functional forms were considered, two without presence of an interaction effect (Scenarios 1 and 2) and four scenarios considering different shapes of interaction terms (Scenarios 3 to 6). All scenarios are visualized in Figure 1, showing the hazard rates used for simulation of the event times in dependence of the biomarker value (dashed black and solid grey line and black scale/axis) and the resulting hazard ratios (using a logarithmic scale) between the treatment groups (red line and scale/axis).

**Scenario 1 .**

No associations between treatment and risk for an event and between the biomarker of interest and risk for an event are present; the hazard rate for each individual was set to 1, irrespective of treatment group and biomarker value (Figure 1(a)).

(1) $$\begin{array}{c} \lambda \left(x\;|\;z,T=0\right)=\lambda \left(x\;|\;z,T=1\right)=1, \end{array}$$

where *λ*(*x*) indicates the hazard rate as a function of time. Consequently, the hazard ratio between the groups is 1 for all covariate values, indicating no biomarker-treatment interaction.

(2) $$\begin{array}{c} \text{HR}\left(z\right)=1. \end{array}$$

**Scenario 2 .**

In the second scenario, the hazard rate depends on the value of the biomarker *Z* for both treatment groups, but the hazard ratio between the treatment groups is the same for all biomarker values, so no biomarker-treatment interaction is present (Figure 1(b)).

(3) $$\begin{array}{c} \lambda \left(x\;|\;z,T=0\right)=0.5\;\exp\left({\left(2z-1\right)}^{2}\right),\\ \lambda \left(x\;|\;z,T=1\right)=\exp\left({\left(2z-1\right)}^{2}\right), \end{array}$$

leading to a hazard ratio of two for all values of Z.

(4) $$\begin{array}{c} \text{HR}\left(z\right)=2. \end{array}$$

**Scenario 3 .**

In the third scenario, a true linear interaction (on the log-hazard scale) between the biomarker of interest and treatment is present, leading to a hazard ratio between the treatment groups of one for a biomarker value of *Z*=0 and to a hazard ratio of exp(0.75)=2.12 for a value of *Z*=1.

(5) $$\begin{array}{c} \lambda \left(x\;|\;z,T=0\right)=0.7\;\exp\left(0.5z\right),\\ \lambda \left(x\;|\;z,T=1\right)=0.7\;\exp\left(0.5z+0.75z\right)=0.7\;\exp\left(1.25z\right). \end{array}$$

The hazard ratio increases linearly on a logarithmic scale.

(6) $$\begin{array}{c} \text{HR}\left(z\right)=\text{exp}\left(0.75z\right). \end{array}$$

The scenario is displayed in Figure 1(c)).

**Scenario 4 .**

In the fourth scenario, a true qualitative biomarker-treatment interaction, with a higher risk for an event under treatment *T*=0 as compared to treatment *T*=1 for patients with a small value of *Z* and a higher risk for an event under *T*=1 for individuals with a large value of *Z*, is considered (Figure 1(d)). The hazard ratio is monotonically, but not linearly increasing over the biomarker range.

(7) $$\begin{array}{c} \lambda \left(x\;|\;z,T=0\right)=0.9,\\ \lambda \left(x\;|\;z,T=1\right)=0.35\text{ exp}\left(1.7\sqrt{z}-0.2z^{2}-0.3z\right). \end{array}$$

The qualitative interaction is indicated by a hazard ratio being smaller than one for values of *Z* < 0.424 and larger than one for *Z* > 0.424.

(8) $$\begin{array}{c} \text{HR}\left(z\right)=\frac{0.35\text{ exp}\left(1.7\sqrt{z}-0.2z^{2}-0.3z\right)}{0.9}=0.389\text{ exp}\left(1.7\sqrt{z}-0.2z^{2}-0.3z\right). \end{array}$$

**Scenario 5 .**

In Scenario 5, the risk for an event is similar under both treatments for most of the individuals, but the risk increases under treatment *T*=1 for large values of *Z* (Figure 1(e)).

(9) $$\begin{array}{c} \lambda \left(x\;|\;z,T=0\right)=0.9,\\ \lambda \left(x\;|\;z,T=1\right)=0.9+1.75z^{8}. \end{array}$$

Consequently, the hazard ratio is close to one for small and moderate values of *Z* but increases for large values. For *Z*=1, the hazard ratio reaches a value of 2.94.

(10) $$\begin{array}{c} \text{HR}\left(z\right)=1+\frac{1.75z^{8}}{0.9}. \end{array}$$

**Scenario 6 .**

In the sixth scenario, the hazard ratio for group *T*=0 depending on *Z* follows a U-shape, while the hazard ratio for *T*=1 is inversely U-shaped (Figure 1(f)).

(11) $$\begin{array}{c} \lambda \left(x\;|\;z,T=0\right)=0.75\;\exp\left(0.4{\left(2z-1\right)}^{2}\right),\\ \lambda \left(x\;|\;z,T=1\right)=1.25\;\exp\left(-0.5{\left(2z-1\right)}^{2}\right). \end{array}$$

This setting leads to a qualitative biomarker-treatment interaction with lower risks for an event under *T*=1 for small and large values of *Z* and lower risks under *T*=0, else indicated by an inversely U-shaped hazard ratio over the range of *Z*.

(12) $$\begin{array}{c} \text{HR}\left(z\right)=\frac{5}{3}\exp\left(-0.9{\left(2z-1\right)}^{2}\right). \end{array}$$

#### 2.2.2. Sample Size

In order to evaluate whether properties of the methods under consideration are related to the sample size of the trial, three different settings for sample sizes were chosen. The generated datasets included 250, 500, or 1000 individuals, which appear to be typical sample sizes for randomized clinical trials.

#### 2.2.3. Censoring Distribution

In addition to the sample size, censoring distributions were varied to produce scenarios with different numbers of observed events. Censoring times were drawn from exponential distributions with hazard rates of *λ*_cens_=0.3 or *λ*_cens_=2, respectively, to produce scenarios with censoring proportions of about 25% and about 67%, leading to numbers of about 188, 375, and 750 expected events for scenarios with low amount of censored observations and of 83, 167, and 333 expected events for scenarios with high amount of censored observations.

## 3. Results

**Figure 2:**
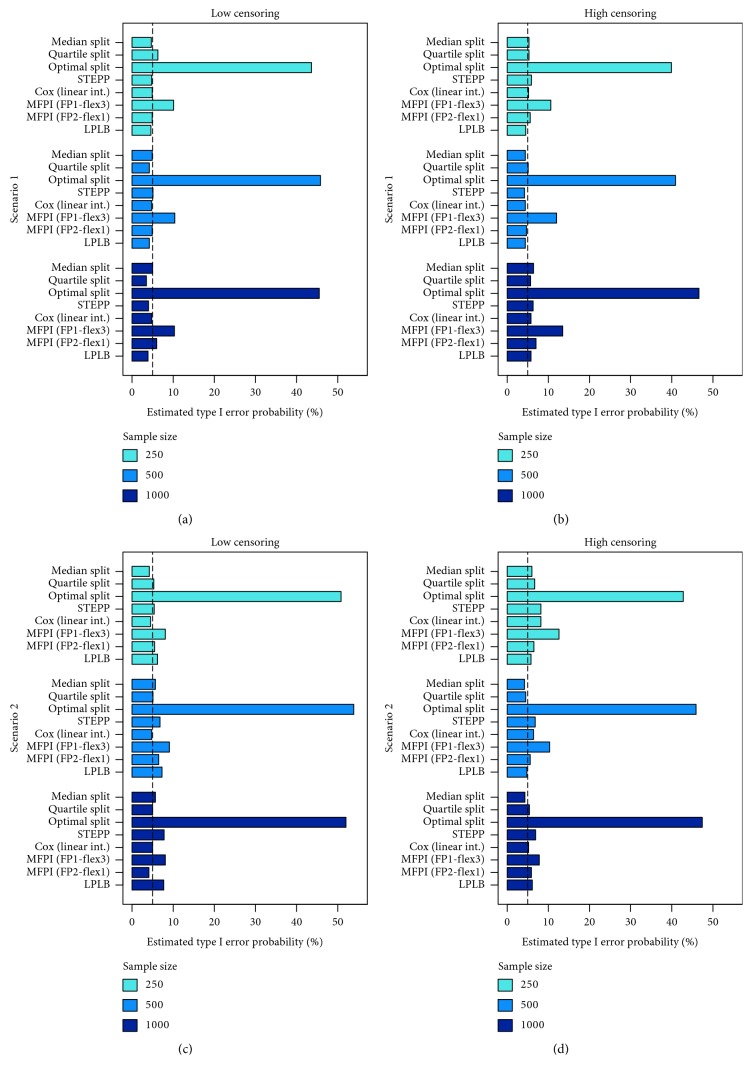
Results of scenarios simulated under the null hypothesis of no biomarker-treatment interaction. Bars represent relative frequencies of falsely rejected null hypotheses.

For each of the 36 scenarios described in Section 2.2, 1000 datasets were generated and the methods presented in Section 2.1 were applied. The *p* value of the corresponding statistical test on biomarker-treatment interaction was saved and compared to the conventional significance level of *α*=5%. Resulting frequencies of type I errors, i.e., proportions of simulated datasets for which a statistically significant biomarker-treatment interaction was found, although it is not present in the corresponding scenario (Scenarios 1 and 2), are shown in Figure 2 for all considered methods and are also tabulated with 95% confidence intervals in Table 1. It can be seen that for the method using the “optimal” cutpoint to define two subgroups to be compared, the probability for a false-positive result was about 50% for both scenarios simulating data under the null hypothesis, irrespective of sample size and amount of censored observations. The Multivariable Fractional Polynomial for Interaction (MFPI) procedure with the FP1-flex3 strategy also provided an increased type I error probability of about 10%. This was mainly caused by those datasets for which different polynomial transformations for the biomarker were selected for models with and without consideration of a biomarker-treatment interaction, leading to a comparison of nonnested regression models. When only simulated datasets were considered, in which the same transformations were used for the models with and without interaction term and consequently two nested models were compared, the estimated type I error probabilities ranged from 3.8% to 6.6%. Contrarily, for datasets with different chosen transformations, the null hypothesis was falsely rejected in 14.1% to 23.8% of the corresponding simulation runs. For the simulations under Scenario 2 with a low sample size of 250 observations and a high amount of censored observations, leading to an expected number of about 83 events, type I error frequencies exceeding the nominal significance level were observed for all methods.

**Figure 3:**
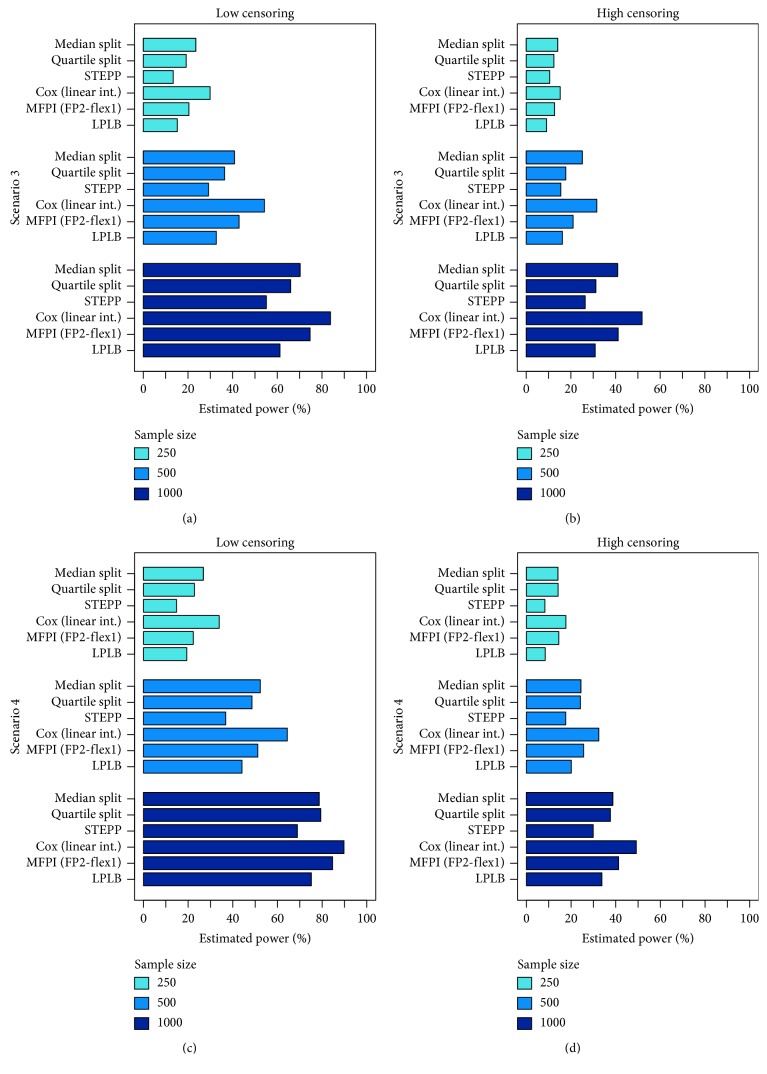
Results of scenarios simulated under the alternative hypothesis of a truly present biomarker-treatment interaction. Bars represent relative frequencies of correctly rejected null hypotheses.

**Figure 4:**
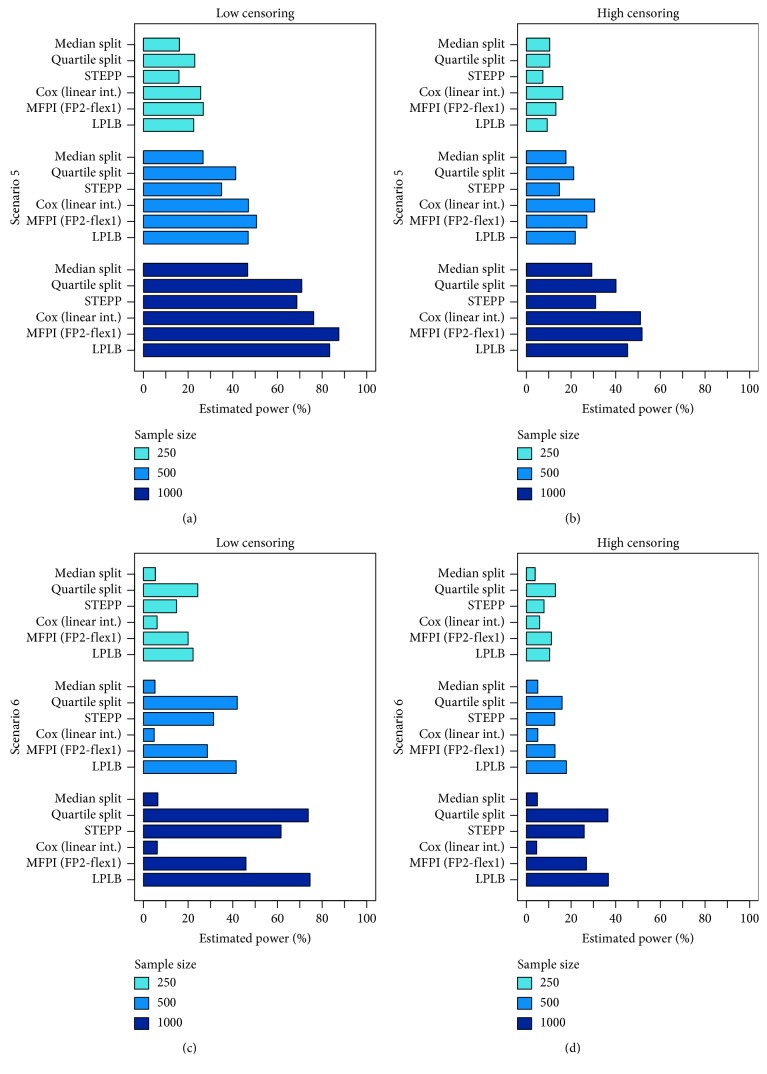
Results of scenarios simulated under the alternative hypothesis of a truly present biomarker-treatment interaction. Bars represented relative frequencies of correctly rejected null hypotheses.

In Figures 3 (Scenarios 3 and 4) and 4 (Scenarios 5 and 6) and in Tables 2 (Scenarios 3 and 4) and 3 (Scenarios 5 and 6), the results of the scenarios with true biomarker-treatment interaction are presented. Consequently, the frequency of rejected null hypotheses can be interpreted as an estimate for the statistical power of the methods under the corresponding settings. As the procedure using two subgroups defined by an optimal, data-driven cutpoint (Optimal split) and the MFPI (FP1-flex3) approach provided type I error probabilities relevantly exceeding the nominal level of *α*=5%, these procedures are not considered in the comparison of statistical power and are consequently not displayed in Figures 3 and 4. Nevertheless, the results are presented in Tables 2 and 3 in italics for completeness.

For the scenario fulfilling the assumption of the standard Cox regression model with a linear interaction term (Scenario 3), the Cox model with linear interaction outperformed all the other investigated methods by achieving the highest observed statistical power (Figures 3(a) and 3(b) and Table 2). The MFPI (FP2-flex1) approach performed slightly better than the approach using two subgroups defined by a split at the median of the variable when the number of expected events was large, but for the scenario with 1000 observations and a low amount of censored observations, the observed power was about 10 percentage points lower for these methods as compared to the Cox regression model with an interaction term considering the biomarker as continuous variable (Cox model with linear interaction: 83.8%; MFPI (FP2-flex1): 74.7%; Median split: 70.2%). The method splitting the data into four subgroups (Quartile split), the STEPP, and the LPLB performed worse than the other approaches.

In Scenario 4, considering a situation with a slightly nonlinear interaction, the Cox regression model considering the continuous biomarker performed best again, followed (at least for scenarios with a large number of events) by the MFPI (FP2-flex1) approach. For small to moderate event numbers, the methods relying on categorization of the data (Median split and Quartile split) performed similarly to MFPI (FP2-flex1). With the chosen settings, the estimated power for LPLB and STEPP was smaller than for the other investigated methods (Figures 3(c) and 3(d) and Table 2).

In the rather complex Scenario 5 with an almost identical risk for an event under both treatments for most patients and an increasing difference between treatments for large values of the biomarker, the MFPI (FP2-flex1) approach performed best in scenarios with a large number of observed events. In scenarios with a high amount of censored observations, the Cox model with linear interaction performed slightly better (small to moderate sample size) or very similar (large sample size) to MFPI (FP2-flex1) (Figures 4(a) and 4(b) and Table 3). When censoring was low and sample size was large, the LPLB approach reached an observed power that was close to MFPI (FP2-flex1) and slightly better than the Cox regression model. While categorization using a Median split was much worse than the other methods for most settings under Scenario 5 (e.g., with an observed power for *n*=1000 and low amount of censored observations of 46.6%), splitting the study population in four subgroups (Quartile split) provided results that were relevantly better than Median split (estimated power for the mentioned settings of 70.9%), but worse than MFPI (FP2-flex1) (87.5%), Cox regression with linear interaction (76.2%), or LPLB (83.4%).

In Scenario 6, the only investigated scenario with nonmonotonous hazard ratio over the range of the biomarker of interest, the Cox model with linear interaction and the procedure defining subgroups at the observed median (Median split) were not able to identify the present biomarker-treatment interaction (estimated power between 4.6% and 6.2% for Cox model with linear interaction and between 3.9% and 6.4% for Median split). The highest empirical power was observed for LPLB and the method defining four subgroups at the observed quartiles (Quartile split). STEPP and MFPI were able to identify the association between biomarker and treatment effect in a relevant amount of generated datasets but performed worse than LPLB and Quartile split (Figures 4(c) and 4(d) and Table 3).

## 4. Discussion

It is well known and accepted that different patients react differently to the same treatment. Consequently, for making a treatment decision, characteristics of the patient or of the disease, e.g., of a tumour, should be considered. Predictive biomarkers, i.e., variables that are associated with the treatment effect, e.g., a hazard ratio between two treatment groups, play an important role for treatment selection. Evidence, whether a biomarker is truly predictive, can only be derived from randomized trials involving patients with different values of the biomarker of interest [8]. In practice, treatment effect heterogeneity over different factors of a categorical variable or over the range of a continuous variable in data collected in a randomized clinical trial is often analysed by the means of subgroup analyses, estimating the treatment effect within patients with similar characteristics and comparing treatment effects between subgroups using a test on interaction [14]. While this procedure is straightforward for categorical variables, it relies on categorization of continuous variables. It was shown for different research questions that categorization leads to a loss of power for detection of true associations [41, 42], and the interpretation of subgroup analyses based on categorized continuous variables was often criticized due to its lack of biological plausibility and its increased chance of spurious findings [17, 18, 43]. One common approach to investigate such interactions between continuous biomarkers and treatment without categorization is the inclusion of the product of the biomarker and the treatment indicator as independent variable in a regression model assuming a linear interaction term. To allow a more flexible modelling of relationships between treatment effects and biomarker values, various methods relaxing the linearity assumption for the interaction term, e.g., the Subpopulation Treatment Effect Pattern Plot (STEPP), the Multivariable Fractional Polynomials for Interaction (MFPI) [19], or the Local Partial-Likelihood Bootstrap (LPLB) [27] approach, were developed.

Comparisons between those methods rarely exist in the literature. Royston and Sauerbrei applied the MFPI and the STEPP method to different datasets [44]. Recently, we investigated the interaction between age and treatment in a randomized trial comparing carotid artery stenting (CAS) to carotid endarterectomy (CEA) for patients with symptomatic, severe carotid artery stenosis (SPACE trial [45, 46]). In this analysis, very similar results were obtained from different methods including Cox regression with linear interaction, STEPP, MFPI, and LPLB [47]. To our best knowledge, only a small number of simulation studies were performed to compare the properties of the different procedures under known scenarios. Royston and Sauerbrei performed a simulation study to compare different MFPI strategies to other regression models and approaches relying on categorization of continuous variables in settings with a continuous outcome [37, 38]. Under all the different MFPI strategies investigated there, the MFPI (FP1-flex3) approach, using one polynomial transformation and allowing for different functional forms in the models with and without considering a covariate-treatment interaction, was identified as the “best” MFPI approach. Bonetti et al. performed a simulation study to evaluate the impact of the parameter settings of the STEPP approach on type I error and statistical power and compared the results to those of a Cox regression model with linear interaction term [32]. Liu et al. also compared performance of their proposed LPLB approach to the Cox regression model with a linear interaction term [27]. Due to the lack of information on the properties of different available methods proposed in the literature for identification of a biomarker-treatment interaction, we performed a simulation study comparing estimates for type I error probability and statistical power of relevant methods under various scenarios. Our aim was to perform a study in the sense of a “neutral” simulation study as described in [48] as we do not favour any of the investigated methods and were not involved in the development or publication of any of them.

As to be expected, we observed that the procedure using an optimal cutoff value determined by maximizing the Wald statistic of the interaction term between the dichotomized biomarker of interest and treatment in a Cox regression model for definition of the subgroups leads to a tremendously increased type I error probability of about 50%. This was observed similarly in simulations presented by Altman et al. who investigated the naïve use of minimum *p* value categorization of a potentially prognostic variable [49]. Interestingly, an increased type I error probability of about 10% in both scenarios with data simulated under the null hypothesis was also observed for the MFPI (FP1-flex3) approach irrespective of sample size and censoring distribution. This was caused by datasets for which different transformations were selected for the models with and without an interaction term. In the simulation study by Royston and Sauerbrei [37], no relevant increase in the probability of false-positive findings was identified for the MFPI (FP1-flex3) approach for most of their investigated scenarios with observed relative frequencies of type one errors ranging from 5% to 7%. Only for scenarios with complex functional forms and a covariate of interest following a skewed distribution (called “badly behaved distribution of *x*” in [37]), an increased type I error probability of up to 20% was found. Maybe this problem is less pronounced in a linear regression setting with quantitative outcome than for our investigated time-to-event endpoint. The originally proposed MFPI (FP2-flex1) approach did not lead to an increased probability of false-positive results and performed generally well for all scenarios. While it was superior to all other methods in a scenario with a hazard ratio constant over a wide range of the biomarker and increasing for individuals with large values when the number of events was large, it was slightly less efficient than a Cox regression model with a linear interaction term in the presence of a truly linear or close to linear biomarker-treatment interaction. Generally, the Cox regression model with a linear interaction term performed better than the other investigated methods for many scenarios. It provided an acceptable probability of false-positive results and higher statistical power than all other methods in the scenario with a truly linear interaction. For small to moderate event numbers, the Cox regression model also outperformed the other methods in scenarios with nonlinear monotonous interaction effects. In one scenario with data generated to provide a nonmonotonous interaction effect over the range of the biomarker of interest, the Cox regression model assuming a linear interaction term was not able to detect this association. For the LPLB procedure, type I error frequencies did not exceed the nominal significance level relevantly and adequate statistical power as compared to the other methods was observed for scenarios with complex functional form of the hazard ratio over the biomarker range. The procedure splitting the data into two subgroups (Median split) led to decreased power for most scenarios, which was also described for other research questions dealing with categorization of continuous covariates [41, 42]. For complex associations, the split into a small number of subgroups might be an adequate first step for data exploration, which was also recommended in the EMA guideline on subgroup analyses [14], or might be used for verification of nonlinear associations found by a corresponding method as also recommended in [38].

Our simulation study has several limitations. Due to limited time and space, only a small number of different scenarios could be investigated. We considered two scenarios in which data were generated under the null hypothesis of no biomarker-treatment interaction and four settings with true biomarker-treatment interactions of different shapes. Additionally, we varied the sample size and used two different amounts of censored observations. We did not vary further aspects of the data generating process as the distribution of the covariate of interest or the influence of further covariates. While some of the methods as fitting a Cox regression model with linear interaction to the data or application of the MFPI approach do not rely on the specification of tuning parameters, other methods such as STEPP or LPLB allow a greater level of user involvement by letting the applicant choose, e.g., the size of the subgroups or the number of overlapping individuals in STEPP or the number of points used for local estimation and the bandwidth in LPLB. As we only used one setting for each of the methods as described in Section 2.1, our findings are only valid for these specific choices, but might not transfer to the methods in general. Further simulation studies are needed to investigate the role of the different tuning parameters on the performance of these methods. In practical applications, subject knowledge could allow more adequate specifications, which might improve performance of the methods compared to our fixed settings. Additionally, we only investigated one potential predictive biomarker and treated it as if investigation of interaction of that biomarker with treatment was the prespecified primary research question. In practice, these kinds of analyses will often be performed as exploratory secondary or add-on analyses, potentially involving multiple biomarkers of interest, and multiplicity issues typically evolving in these situations will have to be addressed adequately. If testing the interaction between a predefined biomarker and treatment is of major interest, this has to be considered in the planning phase of a clinical trial and consequently in the sample size calculation, as often a large sample size is necessary to detect biomarker-treatment interactions [50].

It has to be considered that our simulation study only aims at detection of biomarker-treatment interactions. According to Chen et al., three steps are needed to establish a predictive biomarker in clinical practice: identification of a biomarker, selection of adequate subgroups for treatment stratification, and assessment of clinical utility. Consequently, after identification of a predictive biomarker, subgroups that should be treated by different treatment options have to be identified. For continuous biomarkers, this could be achieved by either application of classification techniques [51] or by exploring the pattern of the treatment effect estimate over the range of the biomarker value. Intuitive visualization as provided by STEPP or by the “treatment effect plot” [52] of the MFPI procedure can be helpful. Additionally, further aspects such as potential risks, patient acceptance, and costs have to be taken into account. Clinical utility might be investigated by randomized clinical trials using biomarker-stratified or biomarker-strategy designs as described by Ondra et al. [53].

As a conclusion of our simulation study, we recommend to perform more detailed and sophisticated analyses for detection of biomarker-treatment interactions than the commonly performed subgroup analyses involving dichotomization of continuous variables. Cox regression models considering linear interaction terms will increase the probability for detection of true interactions as compared to the use of dichotomized variables in many applications. Methods developed for detection of nonlinear interactions can help to identify predictive biomarkers in the presence of complex patterns. We believe that better use of available statistical methods will help to identify and establish predictive biomarkers and increase the number, up to now limited [54], of biomarkers used in clinical practice for treatment stratification and consequently help to improve health care for individual patients.

## Figures and Tables

**Table 1:** Estimated type I error probabilities with exact 95% confidence intervals (in brackets) for Scenarios 1 and 2 for all investigated methods.

		*n* = 250		*n* = 500		*n* = 1000	
		Low cens.	High cens.	Low cens.	High cens.	Low cens.	High cens.
Scenario 1	Median split	4.7%	5.3%	4.9%	4.4%	5.0%	6.4%
		(3.5–6.2%)	(4.0–6.9%)	(3.6–6.4%)	(3.2–5.9%)	(3.7–6.5%)	(5.0–8.1%)
	Quartile split	6.3%	5.3%	4.2%	5.1%	3.5%	5.7%
		(4.9–8.0%)	(4.0–6.9%)	(3.0–5.6%)	(3.8–6.7%)	(2.4–4.8%)	(4.3–7.3%)
	Optimal split	43.6%	39.9%	45.8%	40.9%	45.5%	46.6%
		(40.5–46.7%)	(36.8–43.0%)	(42.7–48.9%)	(37.8–44.0%)	(42.4–48.6%)	(43.5–49.7%)
	STEPP	4.8%	5.9%	5.1%	4.2%	4.0%	6.3%
		(3.6–6.3%)	(4.5–7.5%)	(3.8–6.7%)	(3.0–5.6%)	(2.9–5.4%)	(4.9–8.0%)
	Cox (linear int.)	4.9%	5.2%	4.8%	4.4%	4.8%	5.8%
		(3.6–6.4%)	(3.9–6.8%)	(3.6–6.3%)	(3.2–5.9%)	(3.6–6.3%)	(4.4–7.4%)
	MFPI (FP1-flex3)	10.1%	10.6%	10.4%	12.0%	10.3%	13.5%
		(8.3–12.1%)	(8.8–12.7%)	(8.6–12.5%)	(10.1–14.2%)	(8.5–12.4%)	(11.4–15.8%)
	MFPI (FP2-flex1)	4.9%	5.6%	4.9%	4.7%	6.0%	7.0%
		(3.6–6.4%)	(4.3–7.2%)	(3.6–6.4%)	(3.5–6.2%)	(4.6–7.7%)	(5.5–8.8%)
	LPLB	4.6%	4.5%	4.2%	4.4%	3.9%	5.8%
		(3.4–6.1%)	(3.3–6.0%)	(3.0–5.6%)	(3.2–5.9%)	(2.8–5.3%)	(4.4–7.4%)

Scenario 2	Median split	4.2%	6.0%	5.7%	4.2%	5.7%	4.3%
		(3.0–5.6%)	(4.6–7.7%)	(4.3–7.3%)	(3.0–5.6%)	(4.3–7.3%)	(3.1–5.7%)
	Quartile split	5.3%	6.7%	5.1%	4.5%	5.0%	5.4%
		(4.0–6.9%)	(5.2–8.4%)	(3.8–6.7%)	(3.3–6.0%)	(3.7–6.5%)	(4.1–7.0%)
	Optimal split	50.8%	42.8%	53.9%	45.9%	52.0%	47.4%
		(47.7–53.9%)	(39.7–45.9%)	(50.8–57.0%)	(42.8–49.0%)	(48.9–55.1%)	(44.3–50.5%)
	STEPP	5.4%	8.2%	6.8%	6.8%	7.8%	6.9%
		(4.1–7.0%)	(6.6–10.1%)	(5.3–8.5%)	(5.3–8.5%)	(6.2–9.6%)	(5.4–8.7%)
	Cox (linear int.)	4.5%	8.2%	4.8%	6.4%	5.0%	5.2%
		(3.3–6.0%)	(6.6–10.1%)	(3.6–6.3%)	(5.0–8.1%)	(3.7–6.5%)	(3.9–6.8%)
	MFPI (FP1-flex3)	8.1%	12.6%	9.1%	10.3%	8.1%	7.8%
		(6.5–10.0%)	(10.6–14.8%)	(7.4–11.1%)	(8.5–12.4%)	(6.5–10.0%)	(6.2–9.6%)
	MFPI (FP2-flex1)	5.5%	6.5%	6.5%	5.6%	4.1%	5.9%
		(4.2–7.1%)	(5.1–8.2%)	(5.1–8.2%)	(4.3–7.2%)	(3.0–5.5%)	(4.5–7.5%)
	LPLB	6.2%	5.8%	7.3%	4.8%	7.7%	6.1%
		(4.8–7.9%)	(4.4–7.4%)	(5.8–9.1%)	(3.6–6.3%)	(6.1–9.5%)	(4.7–7.8%)

**Table 2:** Estimated power with exact 95% confidence intervals (in brackets) for Scenarios 3 and 4 for all investigated methods.

		*n* = 250		*n* = 500		*n* = 1000	
		Low cens.	High cens.	Low cens.	High cens.	Low cens.	High cens.
Scenario 3	Median split	23.5%	14.1%	40.8%	25.1%	70.2%	40.9%
		(20.9–26.3%)	(12.0–16.4%)	(37.7–43.9%)	(22.4–27.9%)	(67.3–73.0%)	(37.8–44.0%)
	Quartile split	19.2%	12.4%	36.4%	17.7%	66.0%	31.2%
		(16.8–21.8%)	(10.4–14.6%)	(33.4–39.5%)	(15.4–20.2%)	(63.0–68.9%)	(28.3–34.2%)
	Optimal split	*71.4%*	*57.6%*	*86.4%*	*71.7%*	*97.1%*	*84.6%*
		*(68.5–74.2%)*	*(54.5–60.7%)*	*(84.1–88.5%)*	*(68.8–74.5%)*	*(95.9–98.0%)*	*(82.2–86.8%)*
	STEPP	13.4%	10.5%	29.2%	15.5%	55.1%	26.4%
		(11.3–15.7%)	(8.7–12.6%)	(26.4–32.1%)	(13.3–17.9%)	(52.0–58.2%)	(23.7–29.2%)
	Cox (linear int.)	29.9%	15.2%	54.2%	31.6%	83.8%	51.9%
		(27.1–32.8%)	(13–17.6%)	(51.1–57.3%)	(28.7–34.6%)	(81.4–86.0%)	(48.8–55.0%)
	MFPI (FP1-flex3)	*30.2%*	*18.2%*	*54.2%*	*32.5%*	*82.8%*	*51.1%*
		*(27.4–33.2%)*	*(15.9–20.7%)*	*(51.1–57.3%)*	*(29.6–35.5%)*	*(80.3–85.1%)*	*(48–54.2%)*
	MFPI (FP2-flex1)	20.4%	12.7%	42.9%	21.0%	74.7%	41.2%
		(17.9–23.0%)	(10.7–14.9%)	(39.8–46.0%)	(18.5–23.7%)	(71.9–77.4%)	(38.1–44.3%)
	LPLB	15.2%	9.1%	32.7%	16.2%	61.2%	30.9%
		(13.0–17.6%)	(7.4–11.1%)	(29.8–35.7%)	(14.0–18.6%)	(58.1–64.2%)	(28.0–33.9%)

Scenario 4	Median split	26.8%	14.1%	52.3%	24.4%	78.7%	38.7%
		(24.1–29.7%)	(12.0–16.4%)	(49.2–55.4%)	(21.8–27.2%)	(76–81.2%)	(35.7–41.8%)
	Quartile split	22.8%	14.2%	48.6%	24.2%	79.4%	37.6%
		(20.2–25.5%)	(12.1–16.5%)	(45.5–51.7%)	(21.6–27.0%)	(76.8–81.9%)	(34.6–40.7%)
	Optimal split	*77.6%*	*57.8%*	*92.3%*	*72.6%*	*99.1%*	*88.8%*
		*(74.9–80.1%)*	*(54.7–60.9%)*	*(90.5–93.9%)*	*(69.7–75.3%)*	*(98.3–99.6%)*	*(86.7–90.7%)*
	STEPP	14.8%	8.3%	36.8%	17.6%	68.9%	29.9%
		(12.7–17.2%)	(6.7–10.2%)	(33.8–39.9%)	(15.3–20.1%)	(65.9–71.8%)	(27.1–32.8%)
	Cox (linear int.)	33.9%	17.7%	64.4%	32.4%	89.8%	49.2%
		(31.0–36.9%)	(15.4–20.2%)	(61.3–67.4%)	(29.5–35.4%)	(87.8–91.6%)	(46.1–52.3%)
	MFPI (FP1-flex3)	*40.4%*	*25.8%*	*72.0%*	*38.8%*	*92.4%*	*56.3%*
		*(37.3–43.5%)*	*(23.1–28.6%)*	*(69.1–74.8%)*	*(35.8–41.9%)*	*(90.6–94.0%)*	*(53.2–59.4%)*
	MFPI (FP2-flex1)	22.3%	14.5%	51.2%	25.6%	84.7%	41.3%
		(19.8–25.0%)	(12.4–16.8%)	(48.1–54.3%)	(22.9–28.4%)	(82.3–86.9%)	(38.2–44.4%)
	LPLB	19.4%	8.4%	44.1%	20.1%	75.2%	33.8%
		(17.0–22.0%)	(6.8–10.3%)	(41.0–47.2%)	(17.7–22.7%)	(72.4–77.8%)	(30.9–36.8%)

Due to increased type I error probabilities, results for Optimal split and MFPI (FP1-flex3) are presented in italics.

**Table 3:** Estimated power with exact 95% confidence intervals (in brackets) for Scenarios 5 and 6 for all investigated methods.

		*n* = 250		*n* = 500		*n* = 1000	
		Low cens.	High cens.	Low cens.	High cens.	Low cens.	High cens.
Scenario 5	Median split	16.1%	10.4%	26.7%	17.7%	46.6%	29.3%
		(13.9–18.5%)	(8.6–12.5%)	(24.0–29.6%)	(15.4–20.2%)	(43.5–49.7%)	(26.5–32.2%)
	Quartile split	23.0%	10.5%	41.3%	21.2%	70.9%	40.1%
		(20.4–25.7%)	(8.7–12.6%)	(38.2–44.4%)	(18.7–23.9%)	(68.0–73.7%)	(37.0–43.2%)
	Optimal split	*79.7%*	*60.2%*	*93.8%*	*78.3%*	*99.4%*	*91.9%*
		*(77.1–82.2%)*	*(57.1–63.2%)*	*(92.1–95.2%)*	*(75.6–80.8%)*	*(98.7–99.8%)*	*(90.0–93.5%)*
	STEPP	15.9%	7.4%	35.0%	14.8%	68.7%	31.0%
		(13.7–18.3%)	(5.9–9.2%)	(32.0–38.0%)	(12.7–17.2%)	(65.7–71.6%)	(28.1–34.0%)
	Cox (linear int.)	25.6%	16.3%	47.0%	30.6%	76.2%	51.1%
		(22.9–28.4%)	(14.1–18.7%)	(43.9–50.1%)	(27.8–33.6%)	(73.4–78.8%)	(48.0–54.2%)
	MFPI (FP1-flex3)	*39.7%*	*22.6%*	*67.3%*	*39.7%*	*93.8%*	*66.5%*
		*(36.7–42.8%)*	*(20.0–25.3%)*	*(64.3–70.2%)*	*(36.7–42.8%)*	*(92.1–95.2%)*	*(63.5–69.4%)*
	MFPI (FP2-flex1)	26.8%	13.2%	50.6%	27.1%	87.5%	51.8%
		(24.1–29.7%)	(11.2–15.5%)	(47.5–53.7%)	(24.4–30.0%)	(85.3–89.5%)	(48.7–54.9%)
	LPLB	22.5%	9.3%	46.9%	21.9%	83.4%	45.3%
		(19.9–25.2%)	(7.6–11.3%)	(43.8–50.0%)	(19.4–24.6%)	(80.9–85.7%)	(42.2–48.4%)

Scenario 6	Median split	5.3%	3.9%	5.1%	5.1%	6.4%	4.9%
		(4.0–6.9%)	(2.8–5.3%)	(3.8–6.7%)	(3.8–6.7%)	(5.0–8.1%)	(3.6–6.4%)
	Quartile split	24.3%	13.0%	42.0%	16.0%	73.8%	36.5%
		(21.7–27.1%)	(11.0–15.2%)	(38.9–45.1%)	(13.8–18.4%)	(71.0–76.5%)	(33.5–39.6%)
	Optimal split	*73.8%*	*56.5%*	*88.1%*	*67.1%*	*97.6%*	*86.3%*
		*(71.0–76.5%)*	*(53.4–59.6%)*	*(85.9–90.0%)*	*(64.1–70.0%)*	*(96.4–98.5%)*	*(84.0–88.4%)*
	STEPP	14.8%	7.9%	31.4%	12.7%	61.6%	25.9%
		(12.7–17.2%)	(6.3–9.7%)	(28.5–34.4%)	(10.7–14.9%)	(58.5–64.6%)	(23.2–28.7%)
	Cox (linear int.)	6.1%	5.9%	4.8%	5.1%	6.2%	4.6%
		(4.7–7.8%)	(4.5–7.5%)	(3.6–6.3%)	(3.8–6.7%)	(4.8–7.9%)	(3.4–6.1%)
	MFPI (FP1-flex3)	*23.0%*	*16.5%*	*30.6%*	*18.5%*	*50.9%*	*27.3%*
		*(20.4–25.7%)*	*(14.3–18.9%)*	*(27.8–33.6%)*	*(16.1–21.0%)*	*(47.8–54.0%)*	*(24.6–30.2%)*
	MFPI (FP2-flex1)	20.0%	11.2%	28.6%	12.8%	45.9%	26.9%
		(17.6–22.6%)	(9.3–13.3%)	(25.8–31.5%)	(10.8–15.0%)	(42.8–49.0%)	(24.2–29.8%)
	LPLB	22.2%	10.4%	41.5%	17.9%	74.6%	36.7%
		(19.7–24.9%)	(8.6–12.5%)	(38.4–44.6%)	(15.6–20.4%)	(71.8–77.3%)	(33.7–39.8%)

Due to increased type I error probabilities, results for Optimal split and MFPI (FP1-flex3) are presented in italics.

## Data Availability

All findings are based on simulated data. R code for data generation can be obtained from the corresponding author upon reasonable request.

## References

[1] Thunnissen E., van der Oord K., Den Bakker M. (2014). Prognostic and predictive biomarkers in lung cancer. a review. *Virchows Archiv*.

[2] Robinson W. H., Mao R. (2016). Biomarkers to guide clinical therapeutics in rheumatology?. *Current Opinion in Rheumatology*.

[3] Hoefflin R., Geißler A.-L., Fritsch R. (2018). Personalized clinical decision making through implementation of a molecular tumor board: a German single-center experience. *JCO Precision Oncology*.

[4] Ahmadzada T., Kao S., Reid G., Boyer M., Mahar A., Cooper W. (2018). An update on predictive biomarkers for treatment selection in non-small cell lung cancer. *Journal of Clinical Medicine*.

[5] Ballman K. V. (2015). Biomarker: predictive or prognostic?. *Journal of Clinical Oncology*.

[6] Pritzker K. P. (2015). Predictive and prognostic cancer biomarkers revisited. *Expert Review of Molecular Diagnostics*.

[7] Polley M.-Y. C., Freidlin B., Korn E. L., Conley B. A., Abrams J. S., McShane L. M. (2013). Statistical and practical considerations for clinical evaluation of predictive biomarkers. *JNCI Journal of the National Cancer Institute*.

[8] Hingorani A. D., Windt D. A. v. d., Riley R. D. (2013). Prognosis research strategy (progress) 4: stratified medicine research. *BMJ*.

[9] Senkus E., Kyriakides S., Ohno S. (2015). Primary breast cancer: ESMO Clinical Practice Guidelines for diagnosis, treatment and follow-up. *Annals of Oncology*.

[10] Cardoso F., Senkus E., Costa A. (2018). 4th ESO-ESMO international consensus guidelines for advanced breast cancer (ABC 4). *Annals of Oncology*.

[11] Besse B., Adjei A., Baas P. (2014). 2nd ESMO Consensus Conference on Lung Cancer: non-small-cell lung cancer first-line/second and further lines of treatment in advanced disease. *Annals of Oncology*.

[12] Chen J. J., Lu T.-P., Chen Y.-C., Lin W.-J. (2015). Predictive biomarkers for treatment selection: statistical considerations. *Biomarkers in Medicine*.

[13] Wang R., Lagakos S. W., Ware J. H., Hunter D. J., Drazen J. M. (2007). Statistics in medicine-reporting of subgroup analyses in clinical trials. *New England Journal of Medicine*.

[14] EMA/CHMP (2014). *Guideline on the Investigation of Subgroups in Confirmatory Trial (Draft)*.

[15] Vach W. (2012). *Regression Models as a Tool in Medical Research*.

[16] Royston P., Sauerbrei W. (2008). *Multivariable Model-Building: A Pragmatic Approach to Regression Analysis Based on Fractional Polynomials for Modelling Continuous Variables*.

[17] Assmann S. F., Pocock S. J., Enos L. E., Kasten L. E. (2000). Subgroup analysis and other (mis)uses of baseline data in clinical trials. *The Lancet*.

[18] Pocock S. J., Assmann S. E., Enos L. E., Kasten L. E. (2002). Subgroup analysis, covariate adjustment and baseline comparisons in clinical trial reporting: current practice and problems. *Statistics in Medicine*.

[19] Royston P., Sauerbrei W. (2004). A new approach to modelling interactions between treatment and continuous covariates in clinical trials by using fractional polynomials. *Statistics in Medicine*.

[20] Tian L., Alizadeh A. A., Gentles A. J., Tibshirani R. (2014). A simple method for estimating interactions between a treatment and a large number of covariates. *Journal of the American Statistical Association*.

[21] Fan J., Lin H., Zhou Y. (2006). Local partial-likelihood estimation for lifetime data. *The Annals of Statistics*.

[22] Strimbu K., Tavel J. A. (2010). What are biomarkers?. *Current Opinion in HIV and AIDS*.

[23] Personalized Medicine Coalition (January 2019). Personalized medicine at FDA: 2017 progress report. http://www.personalizedmedicinecoalition.org/Userfiles/PMC-Corporate/file/PM_at_FDA_2017_Progress_Report.pdf.

[24] Wilson M. K., Collyar D., Chingos D. T. (2015). Outcomes and endpoints in cancer trials: bridging the divide. *The Lancet Oncology*.

[25] Bonetti M., Gelber R. D. (2000). A graphical method to assess treatment-covariate interactions using the Cox model on subsets of the data. *Statistics in Medicine*.

[26] Cox D. R. (1972). Regression models and life-tables. *Journal of the Royal Statistical Society: Series B (Methodological)*.

[27] Liu Y., Jiang W., Chen B. E. (2015). Testing for treatment-biomarker interaction based on local partial-likelihood. *Statistics in Medicine*.

[28] R Core Team (2016). *R: A Language and Environment for Statistical Computing*.

[29] Therneau T. M. (2015). *A Package for Survival Analysis in S*.

[30] Therneau T. M., Grambsch P. M. (2000). *Modeling Survival Data: Extending the Cox Model*.

[31] Fox J., Weisberg S. (2011). *An R Companion to Applied Regression*.

[32] Bonetti M., Zahrieh D., Cole B. F., Gelber R. D. (2009). A small sample study of the STEPP approach to assessing treatment-covariate interactions in survival data. *Statistics in Medicine*.

[33] Yip W.-K., Bonetti M., Cole B. F. (2016). Subpopulation treatment effect pattern plot (STEPP) analysis for continuous, binary, and count outcomes. *Clinical Trials: Journal of the Society for Clinical Trials*.

[34] Lazar A. A., Bonetti M., Cole B. F., Yip W.-K., Gelber R. D. (2016). Identifying treatment effect heterogeneity in clinical trials using subpopulations of events: STEPP. *Clinical Trials*.

[35] Yip W.-K., Lazar A., Zahrieh D. https://CRAN.R-project.org/package=stepp.

[36] Royston P., Altman D. G. (1994). Regression using fractional polynomials of continuous covariates: parsimonious parametric modelling. *Applied Statistics*.

[37] Royston P., Sauerbrei W. (2013). Interaction of treatment with a continuous variable: simulation study of significance level for several methods of analysis. *Statistics in Medicine*.

[38] Royston P., Sauerbrei W. (2014). Interaction of treatment with a continuous variable: simulation study of power for several methods of analysis. *Statistics in Medicine*.

[39] Loughin T. M. (1995). A residual bootstrap for regression parameters in proportional hazards models. *Journal of Statistical Computation and Simulation*.

[40] Zhang S., Chen B. (2016). *lplb: Local Partial Likelihood Bootstrap (LPLB) Test, R Package Version 0.1*.

[41] Cohen J. (1983). The cost of dichotomization. *Applied Psychological Measurement*.

[42] MacCallum R. C., Zhang S., Preacher K. J., Rucker D. D. (2002). On the practice of dichotomization of quantitative variables. *Psychological Methods*.

[43] Naggara O., Raymond J., Guilbert F., Roy D., Weill A., Altman D. G. (2011). Analysis by categorizing or dichotomizing continuous variables is inadvisable: an example from the natural history of unruptured aneurysms. *American Journal of Neuroradiology*.

[44] Royston P., Sauerbrei W. (2009). Two techniques for investigating interactions between treatment and continuous covariates in clinical trials. *The Stata Journal: Promoting communications on statistics and Stata*.

[45] Ringleb P., Allenberg J., Berger J. (2006). 30 day results from the SPACE trial of stent-protected angioplasty versus carotid endarterectomy in symptomatic patients: a randomised non-inferiority trial. *The Lancet*.

[46] Eckstein H.-H., Ringleb P., Allenberg J.-R. (2008). Results of the stent-protected angioplasty versus carotid endarterectomy (SPACE) study to treat symptomatic stenoses at 2 years: a multinational, prospective, randomised trial. *The Lancet Neurology*.

[47] Haller B., Eckstein H.-H., Ringleb P. A., Ulm K. (2019). Investigation of age–treatment interaction in the space trial using different statistical approaches. *Journal of Applied Statistics*.

[48] Boulesteix A.-L., Lauer S., Eugster M. (2013). A plea for neutral comparison studies in computational sciences. *PLoS One*.

[49] Altman D. G., Lausen B., Sauerbrei W., Schumacher M. (1994). Dangers of using “optimal” cutpoints in the evaluation of prognostic factors. *JNCI Journal of the National Cancer Institute*.

[50] Bonetti M., Cole B. F., Gelber R. D. (2008). Another STEPP in the right direction. *Journal of Clinical Oncology*.

[51] Lipkovich I., Dmitrienko A., Ralph B. (2017). Tutorial in biostatistics: data-driven subgroupidentification and analysis in clinical trials. *Statistics in Medicine*.

[52] Royston P., Sauerbrei W. (2008). Interactions between treatment and continuous covariates: a step toward individualizing therapy. *Journal of Clinical Oncology*.

[53] Ondra T., Dmitrienko A., Friede T. (2016). Methods for identification and confirmation of targeted subgroups in clinical trials: a systematic review. *Journal of Biopharmaceutical Statistics*.

[54] Selleck M. J., Senthil M., Wall N. R. (2017). Making meaningful clinical use of biomarkers. *Biomarker Insights*.

